# Determining factors on volunteers' presence in hospital response to disasters and emergencies: a qualitative study

**DOI:** 10.5249/jivr.vol13i2.1583

**Published:** 2021-07

**Authors:** Forouzandeh Jannat, Davoud Khorasani-Zavareh, Kiyoumars Allahbakhshi, Javad Aghazadeh-Attari, Saeedeh Nateghinia, Iraj Mohebbi

**Affiliations:** ^a^ Social Determinants of Health Research Center, Urmia University of Medical Sciences, Urmia, Iran.; ^*b*^ Skull Base Research Center, Loghman Hakim Hospital, Shahid Beheshti University of Medical Sciences, Tehran, Iran.; ^*c*^ Department of Health in Emergencies and Disasters, School of Public Health, Tehran University of Medical Sciences, Tehran, Iran.

**Keywords:** Disasters, Emergencies, Hospital response, Volunteers

## Abstract

**Background:**

Volunteers' presence, as a critical issue in hospital response to disasters and emergencies, helps to readiness and quick response to the phenomena, preventing deaths caused by such incidences. This study aimed to determine the main factors affecting popular volunteers' presence in hospital response to disasters and emergencies in Iran.

**Methods:**

This qualitative study, conducted on 31 semi-structured interviews during the years 2019 and 2020, concerning emergency specialist working in various health organization nationwide between June 2019 and April 2020. The non-structured and semi-structured interviews were adopted to gather concept code and analyzed using Graneheim recommendation method.

**Results:**

Four main categories, identified as effective factors on volunteers' presence in hospital response to disasters and emergencies, including nine subcategories: (1) organizing and managing volunteers (with two sub-categories: (a) calling and registering volunteers, and (b) identifying volunteers' ability); (2) organizational structure (with two sub-categories: (a) operational planning and (b) coordination and communication); (3) training (with two sub-categories: (a) training in disasters and (b) volunteer training); and (4) volunteer challenges (with three sub-categories: (a) volunteer interaction with organizations, (b) volunteer logistics, and (c) volunteering culture).

**Conclusions:**

Exploring effective factors as regards volunteers' presence in hospital response phase in disasters to adopt a policy based on the experiences of managers and heads of health can help planners to provide effective design and implementation. It can scientifically contribute to disaster risk management and hospital emergency response in Iran.

## Introduction

Based on the United Nations Office for Disaster Risk Reduction (UNISDR), 4.4 billion people (64% of the world's population) have been affected by natural disasters, creating 2 trillion$ economic damage and causing nearly 1.3 million deaths worldwide.^1,2^ Disasters (such as earthquake, flood, hurricane, cyclone or typhoon, tornado, or fire) are commonly a sudden accident or a natural catastrophe leading to great damage or loss of life. In disasters and emergencies, hospitals and health centers are the first units providing optimal and timely health services and playing a vital and decisive role in reducing deaths and rescuing the injured.^3^ Disasters cause many challenges including lack of beds, overcrowding in the emergency department (ED), and lack of staff to provide treatment services that its response requires a different kind of planning from the daily operation of hospitals.^4,5^

When a disaster (whether man-made or natural disasters) occurs, emergency management organizations and certain non-profit organizations (NGOs) deal with the crisis based on previous plan. Currently, there are groups in society as spontaneous and independent volunteers who offer assistance when the crisis occurs, regardless of its location.^2,6^ Increasing the human resources by using volunteers is one of the approaches to improve the capacity and responsiveness of the health system during disasters. However, in disasters, lack of proper planning as to the health care volunteers in the health sector is a serious problem, which may lead to overcrowding, unprofessional interventions, decreasing safety, failure in applying rules, using resources as well as losing time.^7^ Iran is known as one of ten disaster-prone countries and the majority of population are at risk of disasters including earthquakes and floods.^7,8^ Disasters in Iran were reported to have caused more than 33000 deaths and affected over 3 million people during 210 disasters from 2000 to 2018.^9,10^ Due to the possibility of damages to the health facilities during disasters, health systems should use their maximum capacity to deliver the maximum services in response phase.^11^ For example, all hospitals and health centers were destroyed during Bam earthquake in Iran in 2003.^12^ Managing the health care volunteers is one of the major challenges of health care system.^13^

Despite having large number of popular volunteers in Iran during disasters, there are no specific rules and regulations to organize and utilize volunteers' power within the health system.^14^ Lack of proper planning during crisis for organizing popular volunteers in the health sector has been one of the main problems of Iranian health system.^15^ To that end, the National Disaster Management Organization (NDMO) was founded in Iran to decrease the injuries related to disasters through developing resistance of critical infrastructures by 2030. Health and Medical Care, as one of main task forces of the organization, provides health services for the affected people. The training and establishing of all grassroots and volunteer grassroots organizations were the main purpose of the organization arranged in four stages of crisis management.^16^ Many Iranian organizations, institutions and agencies are in charge of this issue and many forces are sent to the affected areas in incidents, but what is very important and noteworthy is human resource management, which is provided as relief, health and medical volunteers.^17^ However, Iran has recently had some success in terms of disaster management in the health sector, yet numerous studies in this connection have lacked health planning in accidents and an emphasis on preparedness.^18,19^

In the studies of Khankeh et al., the participants' perceptions and experiences have not sufficiently been considered. Therefore, their main experiences and concerns have remained unclear.^18,20,21^ In this regard, the majority of studies have been concerned with disaster management with volunteers' presence, the views of World Health Organization (WHO) and health managers on disasters; however, focused to a lesser extent on disaster volunteers, who can be a very effective and accessible force at lower cost in disasters. Therefore, the researchers decided to conduct a qualitative study to discover the experiences of disaster managers and volunteers in the field of health and medical education. This research was carried out to study the effective factors on the popular volunteers' presence in the hospital response to disasters and emergencies in Iran.

## Materials and Methods 

This qualitative study, focusing on volunteers' presence in Hospital Response to Disasters and Emergencies, was conducted on the emergency and disasters specialist working in the various health organizations throughout Iran during June 2019 and April 2020. 

**Participants' selection**

In the present study, the sampling was performed through snowball method. All participants had practical experience related to disasters, including the experience of emergency evacuation, psychosocial support in disaster, disaster planning, emergency management coordinator, triage, disaster logistics, and disaster training. Different participants were provided in the study for triangulation. The number of participants was based on saturation principles in that the selection was continued until theoretical saturation. Accordingly, 31 eligible individuals participated nationwide in Iran in this study. The samples were drawn from the emergency specialist including academic experts, hospital managers, International Red Cross and Red Crescent Movement, Ministry of Health and Medical Education, and National Disaster Management Organization, and emergency staff. In addition, volunteers in disaster with practical experience or theoretical knowledge in the field of natural disasters were included. Participants had at least 5 years of disaster management experience, including disaster management experience, clinical experience, volunteer recruitment and organization, training during disasters, decision making or coordinator for emergency managers and disaster, and disaster coordinator.

**Data collection procedure**

The main tool for data collection was face-to-face interviews. At the outset, the structure of the interview was determined based on the objectives of the study. Prior to starting the study, the interview method and its questions were confirmed by experts specialized in accidents and disasters. The questions were designed based on respondents 'statements, education, expectations and motivation. Initially, three interviews were non-structured and then 28 subsequent interviews were semi-structured. This was done for the sake of comparing the gathered information as well as organizing the data collection ^22,23^ At this stage, three open-ended questions were used before beginning the interview. The interviews initially included questions such as: "In your experience, what are the effective challenges on volunteers' presence in the hospital response to disasters and emergencies?"; "What effective solutions can be provided to volunteers' presence in the hospital response to disasters and emergencies?"; "How do you assess the effective factors on volunteers' presence in the hospital response to disasters and emergencies?". They provided more elaboration through asking these cases, giving an example and focusing on details. All new concepts were explored. All interviews and their implementation were conducted in Persian, taking place between June 2019 and April 2020. The criterion for termination was the number of participants based on data saturation.

All interviews were conducted face-to-face over a period of 15 to 65 minutes. In case of ambiguity, a second interview was conducted, which lasted between 12 and 38 minutes, with an average of 20 minutes. At the beginning, the researcher introduced himself/herself, explaining the objectives of the study to the interviewees. In order to ensure accurate and prevent data missing, interview sessions were recorded with MP3 player with the participants' consent. All interviews were conducted by a PhD candidate in Health in Emergency and Disaster.

**Data analysis**

This research was conducted qualitatively through content analysis adopting Graneheim and Lundman approach.^24^ Content analysis, being a flexible method to be used for a wide range of textual data, heavily focuses on data coding. Data were collected directly without prior hypothesis through participants' experiences. Codes, concepts, subcategories as well as dimensions were extracted by an inductive process. In order for the researcher to become familiar with the depth and breadth of the data content, the audio file of the recorded interviews was listened several times and then transcribed. This was followed by active performing their repeated reading (searching for meanings and patterns). For open coding, the text of the interviews was read several times and the main concepts were extracted and recorded as code. 

First, the meaning units and then condense meaning units were identified. In the next step, the codes were labeled on the concepts based on the research question. The codes were placed next to each other in terms of similarities and differences, formed subcategories and finally categories. The entire writings were considered as a unit of analysis. Words, sentences or paragraphs were considered as semantic units. Semantic units were a set of words and sentences that were related to each other in terms of content. These units were summed up according to their content and meanings and placed together. The semantic units, according to the concept hidden in them, reached the level of abstraction and conceptualization and then were coded. At this stage, for clarification of certain answers and further explanation, the second interview was conducted with some samples. The codes were compared with each other in terms of similarities and differences and were classified under more abstract categories with a specific label. By comparing categories and deep and accurate reflection, the hidden content in the data was introduced as a study theme.^24^

**Validity and Reliability**

To ensure the validity and reliability of the study, Lincoln and Guba evaluation methods were employed, which is equivalent to the validity and reliability of quantitative research. To that end and based on this method, four criteria of credibility, transferability, dependability and confirmability are considered for evaluation.^25,26^ To increase data credibility, there was long involvement with data. No pre-framework for data collection and analysis as to the research topic was assumed. Moreover, the findings of the analysis were returned to the participants for accuracy, completeness and consistency with the participants' experiences to ensure the accuracy of the codes and interpretations, and to correct the codes that the participants did not express their opinion. The effective factors on popular volunteers' presence in the hospital response to disasters and emergencies were assessed under close supervision. In order to deeply understand the concepts and prevent superficial and mechanical programming, the programming and classification of concepts were done manually with paper and pencil and then categories and subcategories were included. Interviews continued until they reached a theoretical saturation. The criterion for reaching theoretical saturation was the repetition in the extracted codes.

**Credibility: **The credit of qualitative analysis is provided by allocating sufficient time to the data. Continuous comparison was guaranteed through long-term engagement with participants, continuous comparison of participants' expressions, and understanding of their experiences by the researcher, review of members and peer review. Member check: Continuous comparison is done by continual return to data in the analytical stage, which has a role in creating the major categories and subcategories. The main researcher has been involved in all stages of data collection and analysis.

**Confirmability: **Non-interference with personal taste and impartiality has been promoted in various cases of the research process using the writing of notes and the neutrality of data.

**Transferability: **Detailed descriptions of the whole study process were provided.

**Dependability: **Triangulation in data analysis was done in such a way that the researcher checked all the codes and coding was provided independently.

**Ethical considerations**

Conscious consent was obtained from all participants either in black and white by signing consent form of the interviewee or oral acceptance. They were ensured to preserve anonymity and confidentiality, having the right to withdraw from the study at any time. This study was approved by the Research Ethics Committee of West Azerbaijan University of Medical Sciences (Urmia) with the ethical code IR.UMSU.REC.1398.246.

## Results

Based on the demographic characteristics of the experts in this study, 7 people were female and 24 others were male, aged between 30 and 60, all university-educated holding MSc or PhD. Accordingly, the participants were recruited from university experts, hospital managers, Red Crescent managers, managers of the Ministry of Health and Medical Education, and Popular volunteers in disasters in Iran in 2019-2020. Table 1 presents the demographic characteristics of experts and volunteers in the study. 

**Table 1:** Experts and volunteers' demographic characteristics in the study of exploration of effective factors on popular volunteers' presence in the hospital response to disasters and emergencies.

	Variables	Frequency	Percentage
**Demographic features**	Female	7	22
	Male	24	78
**Age (years)**	30-40	8	26
	41-50	12	39
	51-60	11	35
**Education **	Diploma	2	6
	BSc	6	19
	MA	5	16
	PhD	18	58
**work experience (years)**	5-10	7	23
	11-15	15	48
	>15	9	29
**Organization **	International Red Cross and Red Crescent Movement	6	19.35
	Ministry of Health and Medical Education	4	16.12
	Hospital managers	7	22.57
	National Disaster Management Organization	4	16.12
	Academic experts	4	16.12
	Emergency staffs	6	19.35
**Type of Cooperation**	Volunteers	14	45
	Experts	17	55

**Table 2:** Categories, subcategories and codes of effective factors on popular volunteers' presence in hospital response in disasters and emergencies in Iran.

Category	Sub-category	Example of code
**Organizing and managing volunteers**	**Calling, identifying and registering volunteers**	Calling volunteers based on needs assessment
		The need to register volunteers in a national system
		Calling volunteers based on being local in the area
	**Increasing volunteers' capabilities **	The need to identify and select volunteers before the events
		The need to prepare a volunteering ID card for volunteers
		Managing volunteers' recruitment and retention
		System to promote and encourage volunteers
		The need to identify people based on their abilities
		Investigating volunteers' psychological needs
		Identifying volunteers' needs
		Investigating volunteers' motivation
		Employing and guiding volunteers properly
		Evaluating volunteers' abilities and capabilities
		Monitoring and following up volunteers' voluntary actions
**Organizational structure**	**Developing operational program**	The need to pay attention to volunteer-based activities in the chart of the Ministry of Health
		Establishing volunteer management system
		Developing operational scenarios and monitoring volunteer activities
		Identifying improvable areas of applications
		The need for volunteers' job descriptions for different categories
		Designing organizational structure of the hospital with the volunteers' presence, especially in the field of overcapacity
	**Coordination and communication**	Determining methods of communication with volunteers
		Developing a single program for coordination and communication with volunteers
**Training**	**Developing training program in disasters**	The need to develop a training program based on capability
		The need to use a common language for volunteers activities
		Briefing training of upon arrival for volunteers
	**Training volunteers**	The need for volunteers' free training in response to disasters for empowerment based on the hospital HICS chart
		The need to train volunteers to empower the community
		The need for training in the disaster response phase with different scenarios
		The need for volunteer safety and health training
		Recognizing environmental hazards
	**Volunteers' interaction with organizations**	Weak communication between volunteers and officials
		The need to develop a volunteer communication program with hospitals
		Lack of a single communication system for volunteers
	**Logistics of volunteers**	The need for volunteers' proper preparations
		The need for volunteers' nutrition and welfare facilities
		Lack of volunteers' personal equipment
		The need for volunteer liability insurance
	**Volunteering culture**	
		Uncertainty of volunteers' legal obligations
		Weakness in the volunteer organizing
		Volunteers' language and ethnic heterogeneity
		Misusing volunteers' names in incidents
		Volunteers' confusion in disasters
		Volunteers' invasion of hospitals
		Volunteers' excessive excitement
		The need to recognize environmental hazards in hospitals and districts

**Diagram 1:**
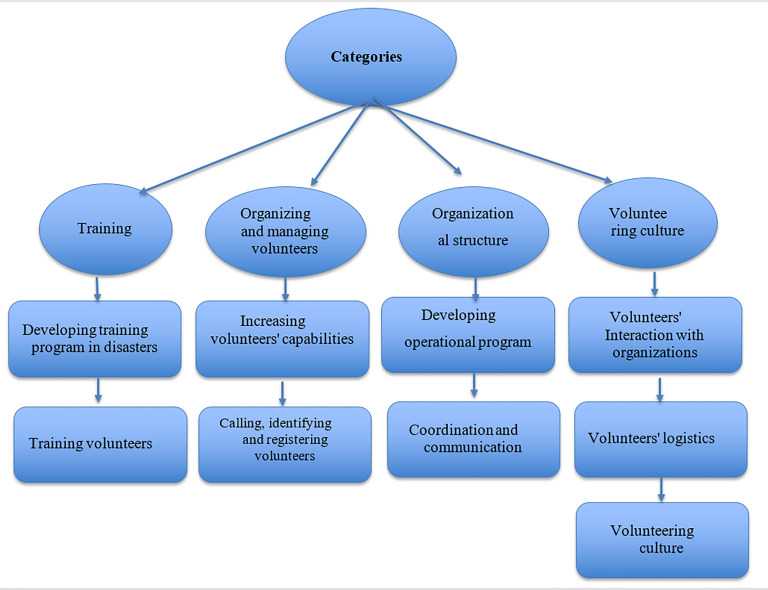
Obtained categories and its Sub-categories on hospital volunteers' presence in disasters and emergencies in Iran.

In the open coding stage, 517 codes were identified and after the initial coding process, concepts and categories were extracted. By performed considerations, four main categories and nine sub-categories were mentioned including (1) organizing and managing volunteers (with two sub-categories: (a) calling and registering volunteers, (b) identifying volunteers' capabilities); (2) organizational structure (with two sub-categories: (a) operational plan, (b) coordination and communication); (3) training (with two sub-categories: (a) training in disaster, (b) volunteer training); and (4) volunteer challenges (with three sub-categories: (a) volunteer interaction with organizations, (b) volunteer logistics, and (c) volunteer culture), as shown in the following table (Table 2) with the factors derived from qualitative analysis (Diagram 1).

**Organizing and Managing volunteers**

***Calling, Identifying and Registering volunteers***

Failing to manage volunteers is one of the effective factors in the response phase, leading to untimely response in disasters and emergencies. To overcome and better organize it, volunteers need to be identified in the area. Health care providers believed that unfamiliarity to disasters and emergency situations causes both local people and volunteers not to be ready for such a time while locals and relatives are the first rescuers or responders in disasters. Volunteers should also be registered according to their ability in the national system, and according to the participants, in order to better identify them, the volunteer's ID card should be issued. Prior to disasters and emergencies, hospitals' manpower needs must be investigated and notified to the volunteer center for registering volunteer forces to be sent to medical centers, if necessary. In this regard, one of the interviewees said: 

*"Providing the necessary documents and identification cards of volunteers is one of the essentials to prevent chaos in critical times. In the meantime, the need to have a plan is felt more than ever. "Despite a clear program for the hospital and the prevention of unexpected challenges, volunteers can be identified and managed." (P16).*

***Increasing the capabilities of volunteers***

Based on participants' experiences, volunteers should be selected before the disasters and categorized according to their interest and ability with special attention to their mental spirits and motivation before disasters. Moreover, volunteers' basic needs must also be examined and pursued to be properly guided and applied. After determining volunteers' capabilities, the necessary training should be proved for them. In addition, practice is one of the most important things that should be repeated periodically. Then the appropriate solution for the ambiguity or problem reported in the practice should be provided to determine the best combination in the operational group and the best role of individuals, so that one can develop new plans. In this regard, follow-up and monitoring voluntary actions should be evaluated. 

*"It is clear that identifying individuals based on their capabilities and the proper use of specialized equipment and personnel will help to manage disasters. The right people need to be put in the right place and at the right time in crises." (P09) "Recruitment, call, training and practice should be in priority. To call and recruit volunteers should be provided in different ways to help" (P18).*

**Organizational Structure**

***Developing an operational plan***

Designing an organizational structure with volunteers' presence, especially in the extra-capacity is a must. Based on the results obtained, the evolving planning process in the healthcare and medical education sectors has been increased in Iran; however, there are still many shortcomings in operational planning. For example, there was no planning to cover all members of the community, accordingly, there is no planning for special groups such as mentally retarded children, people with special illnesses and mental disorder, and the elderly. The planning process should include coordination with other agencies, allocation of agencies, stakeholders and integration with other ongoing planned efforts. Regarding the development of the program, specifying the level of training provided to individuals has been mentioned according to their expertise and skills. Determining the level of equipment in the organization helps to formulate a scenario for crises in the hospital.

*"In the accidents, the influx of people and patients to the hospital increases; and we have to make a plan for search capacity. Procurement and support work should be done by volunteers." (P31) The structure of a separate hospital response should not be considered for volunteers. They should be used in the existing structure, but mechanism of use and organization should be defined to them separately and uniquely. We do not have a specific program for volunteers until we involve people" (P23).*

***Coordination and Communication***

Success in dealing with disasters depends, to a large extent, on coordination and communication in the organizational structure. According to the participants, developing a unified plan to communicate with volunteers and determine communication methods is one of the most important things to respond people. Hospitals should be able to call and manage volunteers according to a common law and find effective ways to communicate with them for practical help when needed. 

*"The hospital is the most complex organization, being always in an emergency condition; and people expect the best services. As a result, hospital communication with volunteers is essential" (P27). Higher organizations should specify administrative rules. But the hospital itself must be autonomous. It must state its needs, prepare its own people in advance, and have common ways of communication with the volunteers". (P31).*

**Training**

***Developing a training program in disasters***

Training of volunteers regarding ability against disasters is one of the effective factors of this research. Training should start from schools and continue to the university and community level. Based on participants' experiences, the training program should be developed and planned at different levels in terms of the capabilities of different groups of volunteers, and all programs should have a common language used in that hospital. Volunteers' scenario-based training program should be planned based on the organizational chart in the hospital, and practiced and repeated periodically to be effective. 

*"Hospitals do not have a plan for hiring volunteers and making instant decisions. They should have a classification in terms of volunteers, level of education and level of service to offer the volunteers. Is the hospital able to classify, train and employ volunteers? Being very important, training volunteers should be based on a common language. The common language in hospitals should be HICS-based, locating them in the HICS boxes based on volunteers' capabilities, literacy, expertise and interest. "(P07)*

***Training of volunteers***

Training factor is one of the facilitators in improving volunteers' skill and efficiency. Providing volunteers with scientific-applied training should be done continuously, completely and in accordance with the latest scientific achievements. Training should be based on volunteers' ability using a common language for their activities. According to participants, volunteers' training should be free, continuous and periodic. This is one of the most important steps to remove the challenges of volunteers' presence disasters. 

*"Identifying the EOC along with monitoring, evaluating and following up on volunteers plays an important role in improving volunteers' training and awareness. Providing pre-disaster training and practice for testing is mentioned."(05P) "Education, being the most important element in disasters, provides the necessary training to defuse a crisis." (P08)*

**Challenges of volunteers**

***Interaction of volunteers with organizations***

Communication in disasters and accidents is an integral part of emergency management, facilitating access and coordination between crisis team members, with implications such as stress reduction and rapid information to achieve disaster management goals. According to participants, there is always a lack of proper communication between volunteers and hospitals as well as the lack of a single communication system for volunteers. The lack of proper communication between public and private hospitals and NGOs is obvious. At present, there are no monitors on organizations to complete and upgrade communication equipment with all organizations and volunteers.

*"There is almost no interaction between people and hospitals. This interaction is three-sided in which people are at one side, the hospital is in another side, and the upstream organizations are in the other. This must be seen from the upstream organizations and communication ways to be informed to the hospitals" (P26)," volunteers can be communicated before, during and after disasters with different means including mobile, SMS and WhatsApp. It must be specified how to communicate and contact the volunteers in each organization."(P01)*

***Logistics of volunteers***

One of the challenges of volunteers' presence in disasters is the lack of provisions, nutrition facilities and personal equipment. This is true with the lack of liability insurance for volunteers' presence in the hospital response phase is an important problem. In addition, the lack of a volunteering ID card and task assignment are logistical challenges of volunteers. There is no certain plan for replacing volunteers with long hours of service since logistics planning is done for all rescue workers. In one unit, a specific program, facilities and support for the volunteers should be seen for several days, and even an alternative program to provide their services should be specified. 

According to participants, rescue teams are present in disasters without a pre-determined plan for volunteers. Meanwhile, ambiguity in emergency operations and insufficient facilities and equipment lead to more confusion and chaos in disasters. 

*"Volunteers should be provided with safety and security, accommodation and food on a shift basis. They need food and clothing. They want drinking water and ... and we must monitor them." (P30), "We must plan volunteers' provision to apply based on experience and capability in the first phase and to see their basic needs for at least 72 hours. We must consider alternatives for this group, like other groups, and provide them with all the basic facilities." (P04)*

***Volunteering culture***

Volunteering culture Promotion and individuals' participation with capabilities in government programs or private organizations for helping their fellows is one of the signs of socio-cultural growth of any society. Ideally, skilled volunteers are present as part of the communities in which they serve. However, participants stated that volunteers' confusion, overwhelm, excessive excitement, and, in some cases, the misuse of volunteer name is caused by volunteering culture weakness. Another most important problem of volunteers in disaster is the challenge of successive crises. And it happens when uneducated people are present in disasters and a bigger crisis is created with untrained forces. 

*"Our country is suffering from weak volunteering culture since it has not well established yet. Behaving emotionally and spontaneously, people appear extremely nervous. "People decide with emotion, and in crises, even though they know they do not have the necessary training to rescue the injured, they are still present in disasters, impeding professional volunteers' immediate help in disasters." (P20).*

## Discussion

In summary, the most effective factors on volunteers' presence in this study include (a) organizing and managing volunteers; (b) calling, identifying and registering volunteers; (c) developing an action plan; (d) developing an education program in disasters for volunteers; (e) volunteer logistics; and (f) Volunteering culture.

This study emphasizes the importance of organizing and managing volunteers. The importance of headquarters and leadership is consistent with other findings in other studies and even in other settings.^27^ Moreover, volunteer management methods with a focus on creating value in the volunteering experience have had a positive effect on volunteers' retention. In particular, training and creating opportunities for volunteers, selecting and identifying qualified volunteers, as well as adapting volunteers to their appropriate duties are associated with a high retention rate.^27,28^ Likewise, other studies have emphasized the need to organize volunteers' presence in the hospital response phase. Apart from the macro level, it is suggested that the call for volunteers should be in the hospital level and presented to the hospitals in an instruction form and protocol accompanied by job description from the ministry. This requires holding training for hospital staff and managers for inviting and calling volunteers to better organize and increase the level of readiness.

There are different types of volunteers: one is employment volunteers that requires special skills or expertise used and needed specifically in the response phase. Another type is general volunteers that ready to help wherever needed.^29^ The other type includes health system volunteers who involve themselves in health care, from prevention and care programs to treatment and follow-up after treatment.^30^ One of the pillars of fulfilling the missions of Red Crescent Volunteers Organization In Iran is to attract efficient and effective volunteer manpower.^31^ Moreover, the important measures of the Tehran Crisis Prevention and Management Organization have been the formation of volunteer emergency response teams since 2005. It is the neighborhoods that have become known as "sustainable" undertaking the recruitment of popular volunteers^32^ Volunteers' organization, maintenance, call and employment are vitally important. They need to be organized based on type of skills, expertise and exact requirement, evaluation as well as review of manpower and volunteers' management.^27^ Although volunteers' call, registration and identification in disasters were introduced as the other main important issue in this study, no specific program is currently available in Iran for calling, identifying and registering volunteers.

Apart from the need to register and call for volunteers, it is important to maintain relation with volunteers as well. There are several ways to maintain this relation. For example, in a study in Canada, continual respect and appreciation, significant volunteering experiences, and communicating and responsiveness to volunteers were important factors in managing and retaining volunteers consistent with the findings of the present study.^33^ Another study conducted in Iran maintains that incentive tools, technical tools of communication and propaganda, information tools and attention to human relations are important indicators of call and maintenance.^34^ Therefore, the importance of this issue and its management is one of the key factors for calling and retaining volunteers. Particular emphasis is suggested to be placed on giving identity and respect to the volunteers. Furthermore, non-material incentive tools, attention to the concept of identifying volunteers to increase their motivation in hospital response as well as retaining volunteers should be taken into consideration.

Iran, as a disaster-prone country, is suffering from government's effective management, needing volunteers' participation. This increase in participation requires a specific operational plan in disasters for volunteers, which does not currently exist. Emphasis on operational and strategic planning to control disasters and obtain the necessary readiness is essential. Disaster management plans not only require a lot of human and financial resources, but also need to develop plans for disasters.^10^ The results on the necessity of persistent management of volunteers are confirmed by other similar studies.^35-37^ Based on a study conducted in Iran, volunteers create different set of topics in organizations; therefore, it requires different planning for working in an organization.^38^ A comprehensive programme should be implemented by Ministry of Health and Medical Education to increase the capacity of hospital beds with volunteers, making optimal use of these people in disasters. This helps personnel to promote readiness of hospitals in disasters and emergencies.

Failure to provide volunteers with required training has been one of the most important effective factors in relief organizations. The participants are complaining about volunteers' insufficient training in the field of disasters. Such insufficiency even affects their motivation, which is in line with other studies and even provides a gap between educational plan, expectations and motivation.^39-41^ Adequate incentives should be provided for them. Volunteer training is essentially effective to successfully implement all response programs in disasters.^42^ According to a study carried out in Iran, the formation of efficient rescue and relief teams, definition of various projects for groups, based on their ability and expertise, as well as their effective training is a determining factor in managing and controlling the processes and activities of voluntary relief.^43^

On the basis of a study, organizations gain great benefits by using trained volunteers instead of salaried employees. Volunteers, having different backgrounds, skills and perspectives in connection with disasters, can undertake different positions and responsibilities;^44^ Accordingly, managers in the executive and operational area of health are recommended to familiarize volunteers with the organization and its goals through preparing a manual. By way of example, the experience of Covid19 can be used for the training method so that a large number of volunteers can be trained online in absentia, even resulting in organization facilitation. In line with other studies,^45^ filling the gap between volunteers' knowledge and attitudes in the direction and tasks of hospitals should be considered seriously. 

The results of the present study show that another effective factor is volunteers' logistics in disasters in that they have always been at risk. Recognizing and meeting volunteers' needs and financial security has become an important issue. Previous studies in Iran have indicated that volunteers should also be considered as part of the rescue team, as they require special care that, if ignored, could cause serious damage to the care system during disasters.^46,47^ In a study evaluating volunteer nursing staff in Finland, language barriers, low interaction with nurses, organizational problems and insufficient information were participants' main challenges.^48^ Another study divulged that defining appropriate task, providing necessary facilities for them and determining working hours in shifts are the most issues influencing the quality of volunteers' services and even the staff of hospital as well as pre-hospital treatment systems.^49^ However, coordination has always been a major challenge in Iran to manage disasters. ^50^ It is recommended that a written plan for the preparation and replacement of volunteers to be written up to 72 hours after disasters and regularly to be rehearsed and reviewed.

In this regard, the capacity of religious groups, especially mosques, churches, and religious temples is suggested to be used to increase volunteers' participation. To achieve a volunteering culture, it should start from schools and colleges, identifying opportunities, strengths, challenges and weaknesses in the country and conducting more research in this area.

Based on the results of the present study, another effective factor in volunteers' presence in disasters is cultural weakness. According to participants, cultural weakness in volunteering caused chaos and disorder in volunteers' presence in disasters and inadequate training causes further problems. This was confirmed by researches conducted in Iran and other countries.^51-53^ In this regard, the capacity of religious groups, especially mosques, churches, and religious temples is suggested to be used to increase volunteers' participation. To achieve a volunteering culture, it should start from schools and colleges, identifying opportunities, strengths, challenges and weaknesses in the country and conducting more research in this area.

Problems in hospitals as organizations demanding for services increases in disasters; accordingly, proper management of these organizations requires to double in ability and skill. Therefore, it is necessary that each hospital, based on its own resources, facilities, risks and characteristics, have a specific disaster management plan or emergency preparedness plan that ensures the self-sufficiency of the hospital. The unpreparedness of hospitals results in having no plan against attack and managing volunteers. Thus, the hospital operational plan is recommended to be presented to volunteers, informing the public before occurring accidents because training the public and the community is important volunteering culture to be modified. This helps to ask people not to refer the hospital in crises if not needed. As a result, the group of volunteers and specialists can provide better and more services to the real injured.

**Strengths and Weaknesses**

This study examined popular volunteers' presence in the hospital response phase in disasters, which has not been addressed in Iran and other parts of the world. The merit of this study is participants' diversity in interview. Apart from exploring the managers' opinions, volunteers' experiences were also explored, which was from different sections of health system from the highest level of decision-making to volunteers' presence on the scene. Moreover the sample size was selected from the specialist countrywide, which is another benefit of this study. One of the limitations of the study was the small number of articles related to the response of popular volunteers to disasters in medical centers, which was overcome by widening the searching strategy and expanding the search timeline. Another limitation was the difficulty of coordinating interviews with a number of professors and officials in organizations due to their busy schedule. In the latter case, a small number of interviews were not possible during the Covid-19 pandemic in an in-person manner and, as a result, the interviews were conducted via WhatsApp, Skype or telephone.

## Conclusion

The main purpose of this study was to determine factors on volunteers' presence in hospital response to disasters and emergencies on a qualitative study. It is important to know volunteers and their ability for carrying out various operations before disasters, train them, empower them in critical matters, and react quickly and effectively when needed. There is a need for organizing and managing volunteers by calling, registering and identifying their capabilities and organizational structure. This includes developing an operational plan, communication and coordination as well as volunteers' training. Prior to crisis and examining volunteers' challenges (including logistics and cultural weakness), activities before, during and after disasters should be specified. Iranian managers and officials in aid organizations should pay special attention to providing volunteers with online training. This is true with developing relevant programs. The future implication of this study can concern disaster risk management and hospital emergency response not only in Iran but also in many less-resourced settings.
